# Lord Bacon's Sanitary Philosophy
*The Works of Lord Bacon; Latin and English editions. In Sixteen Volumes. By Basil Montagu, Esq. London: 1834.


**Published:** 1856-07

**Authors:** 


### LORD BACON'S SANITARY PHILOSOPHY.*

THERE are no passages in the writings of the father of the inductive philosophy, which point out more strikingly the extent of his genius, his penetration, and his singular originality of thought, than those which convey his ideas as to the end, value, and meaning of sound instruction on the subjects of life, health, and disease. A few extracts from the Baconian philosophy bearing on these points, and remarkable for their terseness and deep insight of nature, cannot but prove acceptable to the sanitary reader.

### *The Regimen of Health.*

"There is a wisdom beyond the rule of physic—a man's own observation; what he finds good and what he finds hurtful, is the best physic to preserve health."

"Beware of sudden change in any great point of diet, and, if necessity enforce it, fit the rest to it; for it is a secret both in nature and the state, that it is safer to change many things than one."

"To be free minded and cheerfully disposed at hours of meat, and of sleep, and of exercise, is one of the best precepts of long lasting."

"Entertain hopes, mirth rather than joy, variety of delights rather than surfeit of them; novelties; studies that fill the mind with splendid and illustrious objects, as histories, fables, and contemplations of nature."

"In sickness, respect health principally; in health, action."

"Physicians are some of them so pleasing and conformable to the humour of the patient, that they press not the true cure of the disease; and some others are so regular in proceeding according to art for the disease, that they respect not sufficiently the condition of the patient. Take one of a middle temper."

### *Death.*

"I have often thought upon death, and I find it the least of all evils. All that which is past is as a dream; and he that hopes or depends upon time coming, dreams waking. So much of our life as we have discovered is already dead; and all those hours which we share, even from the breasts of our mothers, until we return to our grandmother the earth, are part of our dying days, whereof even this is one, and

---

* The Works of Lord Bacon; Latin and English editions. In Sixteen Volumes. By BASIL MONTAGU, Esq. London: 1834.

those that succeed are of the same nature, for we die daily; and as others have given place to us, so we must in the end give way to others.

"I know many wise men that fear to die; for the change is bitter, and flesh would refuse to prove it: besides, the expectation brings terror, and that exceeds the evil. But I do not believe that any man fears to be dead, but only the stroke of death; and such are my hopes, that if heaven be pleased, and nature renew but my lease for twenty-one years more, without asking longer days, I shall be strong enough to acknowledge without mourning, that I was begotten mortal. Virtue walks not in the highway, though she go *per alta;* this is strength and the blood to virtue, to contemn things that be desired, and to neglect that which is feared.

"In my own thoughts, I cannot compare men more fitly to anything than to the Indian fig-tree, which, being ripened to his full height, is said to decline his branches down to the earth, whereof she conceives again, and they become roots in their own stock. So man, having derived his being from the earth, first lives the life of a tree, drawing his nourishment as a plant; and made ripe for death, he tends downwards, and is sowed again in his mother the earth, where he perisheth not, but expects a quickening.

"So we see death exempts not a man from being, but only presents an alteration; yet there are some men (I think) that stand otherwise persuaded. Death finds not a worse friend than an alderman, to whose door I never knew him welcome; but he is an importunate guest, and will not be said nay."

### *Building.*

"Houses are built to live in, and not to look on; therefore let use be preferred before uniformity; except where both may be had. Leave the goodly fabrics of houses, for beauty only, to the enchanted palaces of the poets, who build them with small cost. He that builds a fair house upon an ill seat, committeth himself to prison: neither do I reckon it an ill seat only where the air is unwholesome, but likewise where the air is unequal; as you shall see many fine seats set upon a knap of ground, environed with higher hills round about it, whereby the heat of the sun is pent in, and the wind gathereth as in troughs; so as you shall have, and that suddenly, as great diversity of heat and cold as if you dwelt in several places. Neither is it ill air only that maketh an ill seat; but ill ways, ill markets, and, if you will consult with Momus, ill neighbours. I speak not of many more; want of water, want of wood, shade, and shelter, want of fruitfulness, and mixture of grounds of several natures; want of prospect, want of level grounds, want of places at some near distance for sports of hunting, hawking, and races; too near the sea; too remote; having the commodity of navigable rivers, or the discommodity of their overflowing; too far off from great cities, which may hinder business; or too near them, which lurcheth all provisions, and maketh everything dear; where a man hath a great living laid together; and where he is scanted."

### *Medicine, Curative and Preventive.*

"We divide medicine into three parts, or offices: viz., 1st, the preservation of health; 2nd, the cure of diseases; and 3rd, the prolongation of life. For this last part, physicians seem to think it no capital part of medicine, but confound it with the other two; as supposing, that if diseases be prevented, or cured after invasion, long life must follow of course. But, then, they do not consider that both preservation and cure regard only diseases, and such prolongation of life as is intercepted by them: whence the means of spinning out the full thread of life, or preventing, for a season, that kind of death which gradually steals upon the body by simple resolution, and the wasting of age, is a subject that no physician has treated suitably to its merit."

"A work is wanting upon the cures of reputed incurable diseases, that physicians of eminence and resolution may be encouraged and excited to pursue this matter as far as the nature of things will permit; since to pronounce diseases incurable, is to establish negligence and carelessness, as it were by a law, and screen ignorance from reproach."

"To see the daily labours of physicians in their visits, consultations, and prescriptions, one would think that they diligently pursued the cure, and went directly in a certain beaten track about it; but whoever looks attentively into their prescriptions and directions, will find, that the most of what they do is full of uncertainty, wavering, and irresolution, without any certain view or foreknowledge of the course of the cure. Whereas they should from the first, after having fully and perfectly discovered the disease, choose and resolve upon some regular process or series of cure, and not depart from it without sufficient reason."

"And these are the things wanting in the doctrine of medicine for the cure of diseases; but there still remains one thing more, and of greater use than all the rest: viz., a genuine and active natural philosophy, whereon to build the science of physic."

"Things seem to us preservable either in their own substance or by repair; in their own substance, as a fly, or an ant, in amber; a flower, an apple, etc., in conservatories of snow; or a corps of balsam; by repair, as in flame and mechanic engines. He who attempts to prolong life, must practise both these methods together; for separate, their force is less. The human body must be preserved as bodies inanimate are; again, as flame; and lastly, in some measure as machines are preserved. There are, therefore, three intentions for the prolongation of life: viz., 1, to hinder waste; 2, secure a good repair; and 3, to renew what begins to decay."

*Artificial Medicinal Baths and Springs.*

"And for the preparation of medicines; it seems strange, especially as mineral ones have been so celebrated by chemists, though safer for external than internal use, that nobody hath hitherto attempted any artificial imitations of natural baths and medicinal springs, whilst it is acknowledged that these receive their virtues from the mineral veins through which they pass; and especially since human industry can, by certain separations, discover with what kind of minerals such waters are impregnated, as whether by sulphur, vitriol, iron, etc. And if these natural impregnations of waters are reducible to artificial compositions, it would then be in the power of art to make more kinds of them occasionally, and at the same time to regulate their temperature at pleasure. This part therefore of medicine, concerning the artificial imitation of natural baths and springs, we set down as deficient, and recommend as an easy as well as useful undertaking."

*Winter and Summer Sickness.*

"It is commonly seen that more are sick in the summer and more die in the winter, except it be in pestilent diseases, which commonly reign in summer or autumn. The reason is, because diseases are bred chiefly by heat, but then they are cured most by sweat and purge, which in the summer cometh on or is provoked more easily. As for the pestilent diseases, the reason why most die of them in the summer is, because they are bred most in the summer; for otherwise those that are touched are in most danger in the winter."

*Pestilential Seasons. Epidemics.*

"The general opinion is, that years hot and moist are most pestilent; upon the superficial ground that heat and moisture cause putrefaction. In England it is not found true, for there have been great plagues in dry years. Drought tainteth the waters commonly, and maketh them unwholesome."

"Many diseases, epidemical and others, break out at particular times, and the cause is falsely imputed to the constitution of the air at that time when they break forth or reign, whereas it proceedeth indeed from a preceding sequence and series of the seasons of the year; and therefore Hippocrates in his prognostics doth make good observation upon the diseases that ensue upon the nature of the precedent four seasons of the year."

### *Of Drinking Waters.*

"Judgment may be made of waters by the soil whereupon the water runneth; as pebble is the cleanest and best tasted; and next to that clay water; and thirdly, water upon chalk; fourthly, water upon sand; and, worst of all, upon mud. You may not trust waters that taste sweet, for they are commonly found in rising grounds of great cities, which must needs take in a great amount of filth."

### *The Basis of Sanitary Inquiries.*

The rules of the author of the inductive philosophy on the nature of sanitary duties possess great interest at the present time. For the benefit of "officers of health" everywhere, we shall give some of these rules a free transcription.*

"Inquire diligently of dessication, aerifaction, and consumption of bodies inanimate, and of vegetables, and the ways and processes by which they are done; also of the recovery of bodies to their former freshness after they be once dried and withered. From the inquisition touching bodies inanimate and vegetables, let it pass on to other living creatures besides man.

"Inquire touching the length and shortness of life, with the due circumstances which make most for their long or short lives.

"Inquire touching the length and shortness of life in men according to the ages of the world, the several regions, climates, and places of their nativity and habitation.

"Inquire touching the length and shortness of life in man,

---

* For some excellent remarks on this subject, see Mr. Rumsey's Essays on State Medicine.

according to their races or families, as if it were a thing hereditary; also according to their complexions, constitutions, and habits, their statures, the manner and time of their growth, and the making and composition of their members.

"Inquire touching the length and shortness of life in men, according to the times of their nativity, not astrologically, but as whether they were born in the 7th, 8th, 9th, or 10th months; also whether by night or by day, and in what month of the year.

"Inquire touching the length and shortness of life in men, according to their face, diet, government of their life, exercises, and the like.

"Inquire touching the length and shortness of life in men, according to their studies, their several courses of life, the affections of the mind, and divers accidents befalling them.

"Inquire apart touching those medicines which are thought to prolong life.

"Inquire touching the signs and prognostics of long and short life, those which may be seen and observed even in health, whether they be physiognomical signs or any other.

"Inquire touching those things which conserve the body of man from arefaction and consumption, at least which put off and protract the inclination thereto.

"Inquire touching those things which pertain to the whole process of alimentation (by which the body of man is repaired), that it may be good and with the best improvement.

"Inquire touching those things which purge out old matter and supply with new.

"Inquire touching the point of death, and the porches of death leading thereunto from all parts, so that death be caused by a decay of nature and not by violence.

"To know the character and form of old age, make a collection of all the differences both in the state and functions of the body, betwixt youth and old age, that by them you may observe what it is that produceth such manifold effects; let not this inquisition be omitted.

"Inquire diligently touching the differences in the state of the body, and faculties of the mind in youth and old age; and whether there be any that remain the same without alteration or abatement in old age."

Such are some of the advices of Lord Bacon to sanitary and medical inquirers. They are better left free of all comment. Whoever reading them shall rise uninstructed, or shall feel cheated out of time, has full liberty to blame the transcriber for his pains.

